# Assessment of need for inpatient treatment for mental disorder among female prisoners: a cross-sectional study of provincially detained women in Ontario

**DOI:** 10.1186/s12888-019-2083-x

**Published:** 2019-03-27

**Authors:** Roland M. Jones, Kiran Patel, Alexander I. F. Simpson

**Affiliations:** 1 Centre for Addiction and Mental Health (CAMH) and University of Toronto, Unit 3, 1001 Queen St West, Toronto, M6J 1H4 Canada; 2 Centre for Addiction and Mental Health (CAMH) and University of Toronto, Unit 1, 1001 Queen Street West, Toronto, ON M6J 1H4 Canada

**Keywords:** Prison, Corrections, Mental health, Assessment, Hospital, Women, DUNDRUM

## Abstract

**Background:**

International studies show a consistent finding of women in prisons as having a high prevalence of mental disorder. Most will be treated within the prison however the most severely ill require transfer to a hospital facility. The primary aim of our study was to survey the total provincial female prison population in Ontario, Canada, to determine the proportion that require treatment in a psychiatric hospital, and the security level required. The secondary aim was to investigate the validity and psychometric properties of DUNDRUM-1 and DUNDRUM-2 in making these assessments.

**Methods:**

We carried out a cross-sectional study of all remand and sentenced female inmates detained in all 16 provincial jails that hold women in Ontario. The severity of mental health need was categorised by mental health staff on a five-point scale. Two forensic psychiatrists then examined all medical files of prisoners that had been categorised in the highest two categories and a random sample of nearly a quarter of those in the third category. An overall opinion was then made as to whether admission was required, and whether a high intensity bed was needed, and files were rated using DUNDRUM-1 and DUNDRUM-2.

**Results:**

There were 643 female inmates in provincial prisons in Ontario. We estimated that approximately 43 (6.7%) required admission to a hospital facility, of which 21.6 [prorated] (3.4%) required a high intensity bed such as a psychiatric intensive care bed within a secure hospital. The DUNDRUM-1 and -2 tools showed good internal validity. Total scores on both DUNDRUM-1 and DUNDRUM-2 were significantly different between those assessed as needing admission and those who did not, and distinguished the level of security required.

**Conclusion:**

This is the first study to determine level of need for prison to hospital transfers in Canada and can be used to inform service capacity planning. We also found that the DUNDRUM toolkit is useful in determining the threshold and priorities for hospital transfer of female prisoners.

## Background

International studies show a consistent finding of women in prisons as having a high prevalence of psychiatric disorders including psychotic illnesses, bipolar disorder, personality disorder, and drug dependence [1–3]. Approximately 4% of women in prisons worldwide have a psychotic illness, and around 14% have a major depressive disorder [4]. Moreover, this group frequently have complex and multiple needs due to having simultaneous mental disorders, high rates of previous trauma [5] and high levels of psychological distress [6]. Most women who have identified mental health needs will be treated within the prison however those with the most severe and urgent needs require transfer to a hospital facility. There are multiple barriers to admission and the number of women who are transferred to hospital is likely to be smaller than those who are in need. There have been a small number of controlled studies that have evaluated therapeutic interventions among female offenders, which have mainly been psychological interventions for depression and trauma, carried out in the USA. Meta-analysis of these studies has shown a small but significant benefit of intervention [7]. A recent study of prisoners sampled from two UK prisons assessed prevalence of mental disorder and evaluated whether the prisoners’ mental health treatment needs had been met [8]. The authors found that 80% of female prisoners were in need of some form of treatment, and of those, there was unmet need (defined as when there was a recommended treatment or referral to an appropriate service which had not been made) in just over a half of the women. Although the proportion of women in prison who have a diagnosed mental disorder has been studied in several different countries, and there is some evidence as to overall treatment needs, there has been a particular lack of measurement of the numbers of women that require treatment in hospital. In order to develop inpatient services to meet the needs of female prisoners, there must be an attempt to quantify the level of need, and therefore to estimate the number and proportion of female inmates who are most severely ill, and who need transfer to hospital for treatment. To our knowledge, there has only been one previous study that has directly assessed need for admission for female prisoners. Maden et al. [9] surveyed 301 female sentenced prisoners in the UK selected at random in 1988/9 and found that 5% required hospital admission. No study has evaluated the need for hospital treatment among female remand prisoners, who tend to have higher levels of psychosis than female sentenced prisoners [10]. The DUNDRUM toolkit [11] is a set of five structured professional judgement (SPJ) tools to assist clinicians in making decisions around treatment needs for people with a mental disorder who are referred to, or are under the care of forensic mental health services. It is therefore a needs assessment tool, not a violence risk assessment tool. The DUNDRUM-1 triage security scale is designed to assist in determining the level of hospital security that a person requires (high, medium, low, open facility or out-patient) and among those admitted to hospital, scores have been shown to distinguish between those at every security level [12]. DUNDRUM-2 is designed to assist in determining the urgency of admission to hospital, and has also been shown to distinguish between those who are admitted and those who are not admitted from a hospital waiting list [13] and to have good inter-rater reliability and internal consistency. The other sections in the five-tool suite are DUNDRUM-3 (programme completion), DUNDRUM-4 (recovery scales), and the fifth scale is a patient self-rating scale, which is used to assess a patient’s readiness to move to less secure settings. DUNDRUM-1 and DUNDRUM-2 are therefore potentially useful tools to aid in decision-making around security level and urgency of transfer from prison to hospital. The original validation studies included both males and females (the results for females however were not specifically reported), and there have been no validation studies specifically for female prisoners. As part of a plan for service development in the province, we wished to estimate the hospital bed capacity required to meet the needs of severely mentally ill female inmates. We therefore set out to estimate the number of female prisoners currently in provincial jails in Ontario that require mental health treatment in a hospital facility. The primary aim of our study was to survey the total provincial female prison population in Ontario, Canada to determine the proportion that require treatment in a psychiatric hospital, and the security level required. The secondary aim was to investigate the utility of DUNDRUM-1 and DUNDRUM-2 in making these assessments.

## Methods

### Study design and setting

We carried out a cross-sectional study of females detained in all provincial jails that detain females in Ontario. Ontario is the most populous province in Canada, and has a population of nearly 14.2 million people [14]. Provincial jails in Ontario hold remand prisoners, and those sentenced to less than 2 years in custody. There are 16 jails in Ontario that detain women, with a total population of approximately 650. Inmates who require treatment on an involuntary basis can only receive treatment under the Ontario Mental Health Act in a designated facility, known as a Schedule 1 psychiatric facility. Aside from psychiatric hospitals operated by the Ontario Ministry of Health and Long-Term Care that are designated Schedule 1 facilities, there is also a Schedule 1 psychiatric facility known as a “treatment centre” that is operated by the Ministry of Community Safety and Correctional Services (MCSCS), which is for male prisoners who require psychiatric treatment. There are no such treatment centres for women prisoners, and therefore those who are severely ill or who need involuntary treatment must be transferred to a local Schedule 1 psychiatric facility. The majority of Schedule 1 facilities are for general psychiatric inpatient care and fall below the security level required for remand prisoners and most sentenced prisoners. As a consequence, female prisoners admitted to general psychiatric units are required by MCSCS, to be under the supervision and custody of Corrections Officers at all times. This creates practical difficulties for the treating hospitals, for the correctional institution, and most importantly for the patient needing care and treatment.

### Ethical approval

Research ethical approval was granted by the Centre for Addictions and Mental Health Research Ethics Board (#018/2018).

### Participants

All 643 women who were on remand or sentenced in a provincial jail in Ontario, on 18th July 2017.

### Variables and procedure

All inmates are routinely screened at reception using the Brief Jail Mental Health Screen (BJMHS) [15]. Inmates with identified mental health needs are then referred for further assessment and treatment as necessary to the jail healthcare service (comprising primary medical care, psychiatry, psychology, nursing and social work disciplines). Health care managers at each institution were asked to categorise the severity of mental health need of every prisoner on a 5-point scale as either “Very Serious”, “Serious”, “Moderate”, “Mild” or “None” on 18th July 2017. Two forensic psychiatrists (RJ and KP) then examined all medical files of prisoners that had been categorised in the highest two categories by the healthcare managers (“Very Serious” or “Serious”) as those likely to contain individuals who would be in need of hospital treatment. A random sample of approximately 25% of case files of those in the “Moderate” category were also reviewed to ensure that this group did not contain inmates who might also require admission.

Following review of the case files by either of the two forensic psychiatrists, a clinical assessment was made as to whether the person required treatment in hospital, and if so, whether a high intensity bed was required. A high intensity bed was defined as a psychiatric intensive care bed within a secure hospital in which higher levels of physical security and higher staff to patient ratios are required than for a general bed in a secure hospital. Clinical diagnoses as recorded in the case files were also obtained. Each file was rated using DUNDRUM-1 and DUNDRUM-2 [11]. DUNDRUM-1 is an 11-item scale, each item is rated 0–4, and is designed to assist structured decision making as to the level of security required for someone who needs to be transferred to hospital from the criminal justice system (court or prison), or who has been referred from a community mental health facility for transfer to a more secure hospital. DUNDRUM-2 is a 6-item scale, to assist clinicians in determining the urgency of hospital admission. The individual items of DUNDRUM-1 and DUNDRUM-2 are listed in Table 1.

**Table 1:** DUNDRUM-1 and DUNDRUM-2 scores by category of hospital bed required

	n	Psychotic disorder n(%)	Mood disorder n(%)	Substance Use Disorder n(%)	Anxiety disorder n(%)	DUNDRUM-1 Mean score (SD)	DUNDRUM-2 Mean score (SD)
Admission not required	35	11 (31.4)	7 (20.0)	16 (45.7)	7 (20.0)	12.9 (8.3)	4.1 (3.7)
General secure bed	20	18 (90.0)	14 (70.0)	4 (20.0)	4 (25.0)	17.5 (6.9)	9.9 (4.9)
High Intensity secure bed	11	8 (72.7)	4 (36.3)	1 (9.0)	2 (18.2)	25.1 (4.7)	13.2 (5.3)

A random sample of 10 files was rated by both psychiatrists to assess inter-rater reliability of assessment of need for admission and of DUNDRUM ratings.

### Statistical analysis

We calculated the total number and proportion of women who were assessed as requiring treatment in hospital. We calculated inter-rater reliability by selecting 10 cases randomly which both raters independently rated, and calculated the weighted kappa and percent agreement coefficient for scores. We calculated the receiver operating characteristics area under the curve (AUC) for the DUNDRUM-1 and -2 using both need for admission and need for high intensity bed as outcomes, and DUNDRUM-1 and -2 scores as predictors. For each item we calculated odds ratios for the association between item score and need for admission as the dependent variable to estimate the extent to which each item predicted need for admission. We then calculated odds ratios using the total scores both for DUNDRUM-1 and DUNDRUM-2 to investigate whether there was an association between sub-scale scores and need for hospital admission. Stata 14 was used for all analyses [16].

## Results

There were 643 female inmates in provincial prisons in Ontario, of whom 199 were on remand, 442 were sentenced, and 2 were on immigration hold. The mean age was 34.2 (SD 12.2). There were 13 cases (2.0%) categorised during the assessment by healthcare managers as “Very Serious”, 40 (6.2%) categorised as “Serious” and 67 (10.4%) categorised as “Moderate”. We examined the case files of all in the highest 2 categories; there were 3 missing files, giving a total of 50 cases. We also examined a sample of 16 moderate files (23.9% of all moderates), making 66 in total. We found that of those categorised as “Serious” or “Very Serious”, 25 (50%) were assessed as needing a hospital admission, of which 8 were determined to require a high intensity bed. Of those categorised as “Moderate”, 4 (25%) were assessed to require a hospital admission, 3 of which required a high intensity bed. Given that there were 3 missing files from those identified as “Serious” or “Very Serious” categories and given that we found that 50% of patients in this category needed admission, we estimate that one or two of the missing cases would also need admission. The prevalence of diagnoses recorded by type of admission required as well as the mean DUNDRUM-1 and DUNDRUM-2 scores is shown in Table 1. Taken together, this means that if the sample of case files rated as “Moderate” is representative of all cases rated as “Moderate”, the total prorated number of inmates needing admission rated as “Moderate” would be 16.75, and assuming that no cases rated as “Mild” or “None” would require admission, and that among the three missing files, 1.5 (prorated) would require admission, in total 43.25 inmates (6.7%) need admission, of which approximately 21.6 (3.4%) require a high intensity bed. The Cohen’s Kappa coefficient and Percent Agreement coefficient for need for admission was 0.78 and 0.9 respectively, indicating good agreement. The weighted Cohen’s Kappa coefficient for all DUNDRUM-1 items was 0.59 (95% CI 0.47–0.71) and Percent Agreement Coefficient of 0.90 (95% CI 8.87–0.82). For DUNDRUM-2 the weighted Cohen’s Kappa coefficient for DUNDRUM-2 was 0.43 (95% CI 0.23–0.64), and Percent Agreement Coefficient 0.82 (95% CI 0.77–0.89, indicating moderate agreement [17].

**Fig. 1:**
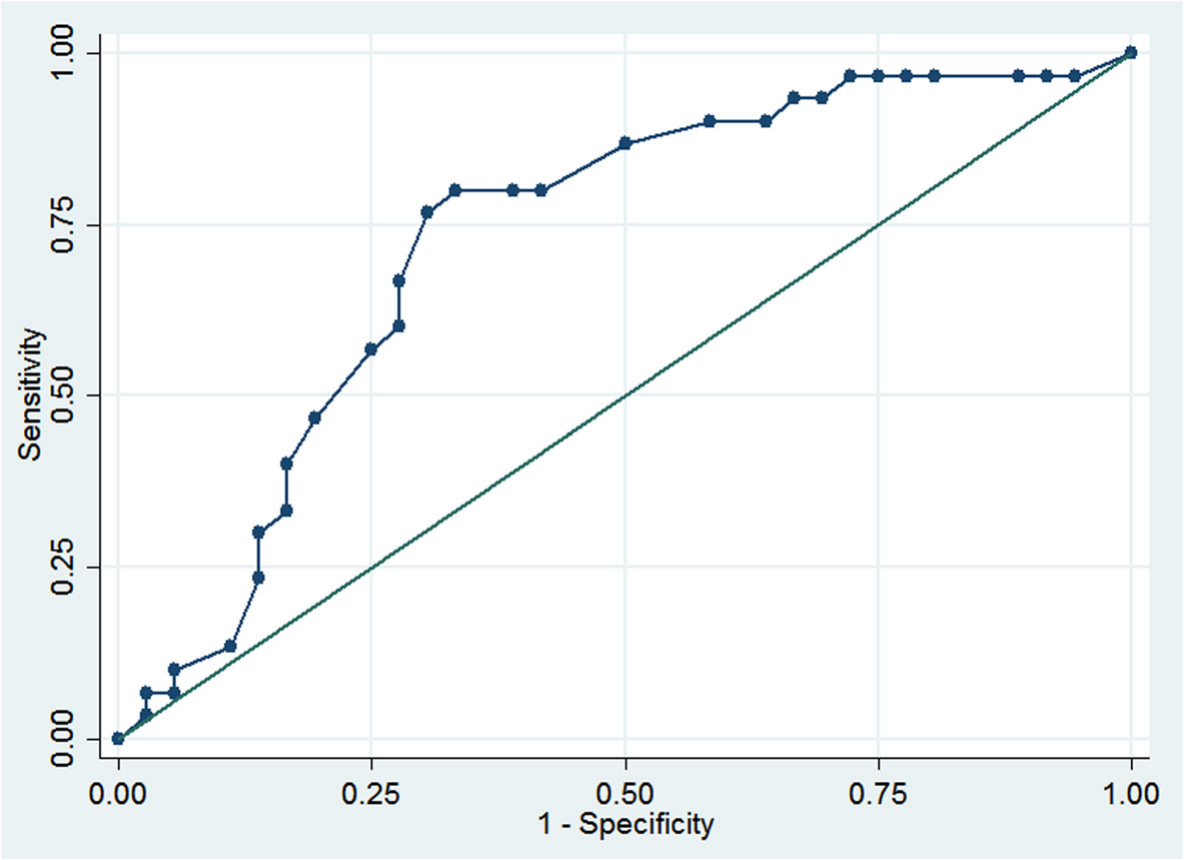
Receiver Operating Characteristics of DUNDRUM-1 Triage Security Score by need for hospital admission. AUC = 0.75

The mean total on the DUNDRUM-1 was 16.3 (SD = 8.5). We calculated the receiver operating characteristics of the total DUNDRUM-1 scores to distinguish need for admission versus no need for admission, needing a general hospital bed versus no admission, and need for a high intensity bed versus a general hospital bed. We found that total DUNDRUM-1 scores distinguished between those needing hospital admission versus those that did not, AUC = 0.75 (95% CI 0.63–0.88, see Fig. 1). We found that DUNDRUM-1 scores for those requiring a high intensity bed were significantly higher than those needing a general hospital bed (using independent samples t-test, t = 3.27, *p* = 0.003, AUC = 0.83, 95% CI 0.67–0.98). We also found that DUNDRUM-1 scores among those that required a general bed were significantly higher than those not requiring admission (t = 2.10, *p* = 0.04), and DUNDRUM-1 scores distinguished between them, AUC = 0.68 (95% CI 0.53–0.82). We calculated odds ratios for each item of the DUNDRUM-1 using need for admission as the dependent variable (see Table 2). We found a significant relationship between total score and need for admission (OR = 1.13, 95% CI 1.05–1.21, *p* < 0.001). When each item was examined individually, we found no relationship between either seriousness of self-harm or immediacy of risk of suicide and need for hospital admission. The AUC for DUNDRUM-1 predicting need for admission was slightly higher when items 2 (seriousness of self-harm) and item 4 (immediacy of risk of suicide) were omitted from the total scores (AUC = 0.82 95%, CI 0.71–93) compared with (AUC = 0.75, 95% CI 0.63–0.88).

**Table 2:** Association between individual items of DUNDRUM-1 and DUNDRUM-2 and need for admission

	OR (95% CI)	p
DUNDRUM-1 items (*n* = 66)		
1 Seriousness of violence	2.07 (1.30–3.30)	0.002
2 Seriousness of self-harm	0.87 (0.57–1.35)	0.546
3 Immediacy of risk of violence	2.68 (1.62–4.43)	< 0.001
4 Immediacy of risk of suicide	0.86 (0.51–1.44)	0.563
5 Specialist forensic need	5.04 (1.83–11.45)	< 0.001
6 Absconding / Eloping	1.95 (1.15–3.30)	0.013
7 Preventing Access	1.66 (0.97–2.84)	0.067
8 Victim Sensitivity	1.60 (0.91–2.82)	0.103
9 Complex needs re violence	2.43 (1.44–4.12)	0.001
10 Institutional behaviour	4.49 (2.17–9.27)	< 0.001
11 Legal Process	1	na
Subtotal	1.13 (1.05–1.21)	0.001
DUNDRUM-2 items (n = 66)		
1 Urgency: Remand / Sentenced prisoner	3.02 (1.77–5.14)	< 0.001
2 Mental Health	7.59 (2.96–19.450	< 0.001
3 Suicide Prevention	0.86 (0.54–1.37)	0.526
4 Humanitarian	4.01 (2.17–7.40)	< 0.001
5 Systemic	3.73 (2.12–6.73)	< 0.001
6 Legal Urgency	1.53 (0.97–2.42)	0.068
Subtotal	1.36 (1.18–1.57)	< 0.001

**Fig. 2:**
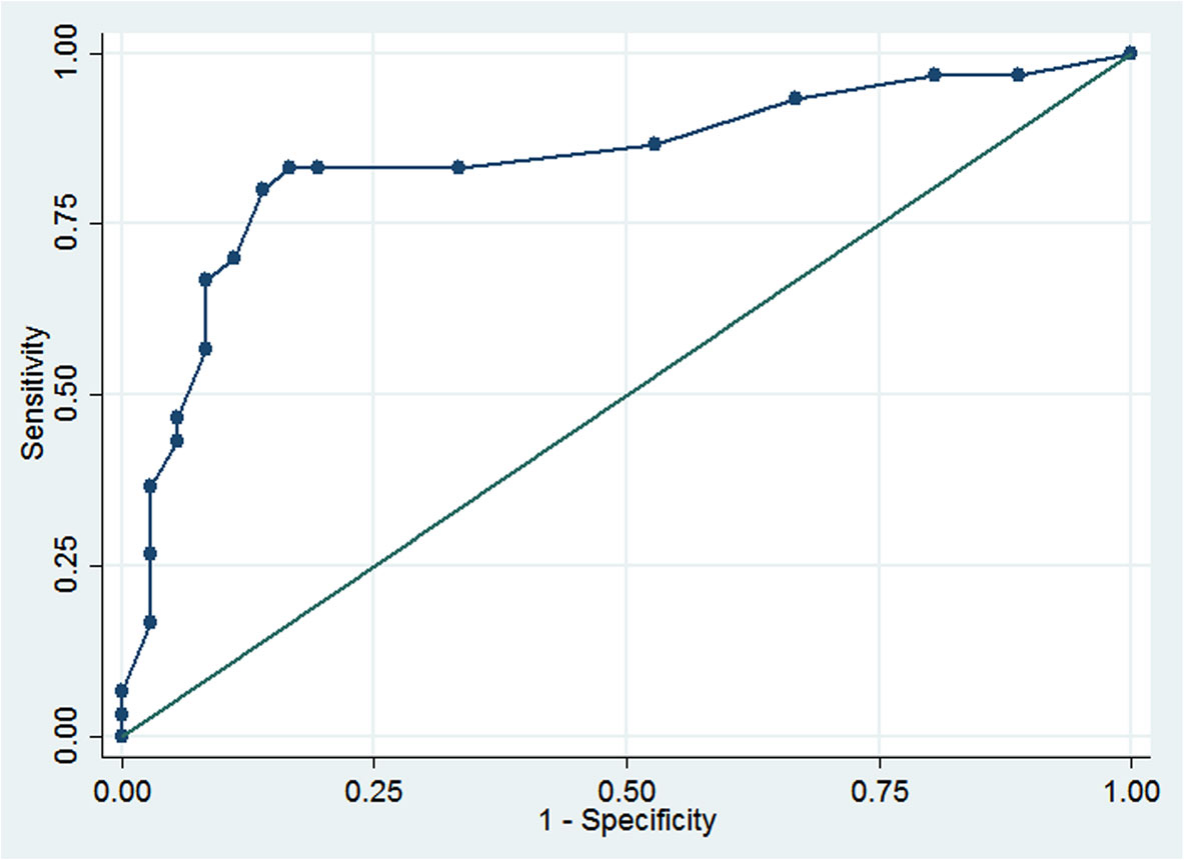
Receiver Operating Characteristics of DUNDRUM-2 Triage Urgency Score by need for hospital admission. AUC = 0.84

The mean total score on the DUNDRUM-2 was 7.38 (SD = 5.62). We investigated receiver operating characteristics of the DUNDRUM-2 to distinguish between need for admission versus no need for admission, between need for general hospital bed versus no admission, and between need for high intensity bed and general hospital bed. We found that DUNDRUM-2 discriminated between need for hospital admission and no admission: AUC = 0.84 (95% CI 0.74–0.94, see Fig. 2), and between need for general hospital admission and no need for admission: AUC = 0.81, (95% CI 0.67–0.94). DUNDRUM-2 did not significantly distinguish between those needing a high intensity bed from those needing a general bed (AUC = 0.6, 95% CI 0.47–0.90). We found a linear relationship between total DUNDRUM-2 scores and need for admission (OR = 1.36, 95% CI 1.18–1.57, *p* < 0.001). Higher scores on each item of DUNDRUM -2 were found to be strongly associated with need for admission, with the exception of Suicide Prevention (OR = 0.86, *p* = 0.525).

## Discussion

We estimated that among a population of 643 provincially detained women in Ontario approximately 43 required admission to a hospital facility, and of those approximately 22 required a high intensity bed. This equates to 6.7% of the female provincially incarcerated population needing to be admitted to a psychiatric hospital for treatment, and 3.3% requiring a high intensity bed. There have been precious few surveys that have estimated the need for hospital treatment for any prison population. Even fewer have been carried out following a clinical assessment from case file review or clinical interviews with inmates [9, 18, 19] as opposed to estimations based on proportions of inmates with mental disorders. Only one of these studies included women prisoners [9]. The studies by Maden et al. [9] and Birmingham et al. [19] both estimated that 3% of male remand prisoners required immediate transfer to hospital. Maden [9] estimated that 5% of female remand prisoners required immediate transfer to hospital, and another 9% required further assessment, some of which may have been determined to require hospital treatment. Our finding of 6.7% requiring admission to hospital is therefore remarkably similar to the figure estimated by Maden, even though that study was carried out in a different country and nearly 30 years earlier. Perhaps little has changed in terms of the community drivers of criminalisation of mentally ill persons, and prison services for people with mental disorders still require advocacy and development. Adequate screening to detect mental disorder in the inmate populations is vital followed by triage and accurate assessment of the level of need and access to appropriate and acceptable services to treat mental illness [20, 21]. Despite few previous studies to have estimated level of need, there have been studies that have reported the number of female prison to hospital transfers in various settings for comparison. Rutherford et al. [22] reported that among over 3300 new female receptions in a prison in UK in 1995, only 1.9% were transferred to hospital during a 12-month period. The authors suggested that this represented a high level of unmet need. Furthermore, a large survey by Bartlett et al. [23] in 2012 reported that 100 women were identified as requiring hospital transfer from a UK prison with capacity of 501 women over a 34-month period. The figure however is not directly comparable to our study as it included women who were directed to hospital by court for assessment or treatment as well as those that needed urgent hospital treatment for mental disorder. Nevertheless, this study highlighted that the number of prisoners that receive inpatient treatment may be lower than those identified as being in need of it as only 86 of the 100 were actually transferred to hospital during the study period, many having been released before they could be admitted. Our estimate of need is therefore higher than the actual transfer rate in the UK, but similar to the level of need previously estimated. We found that higher scores on DUNDRUM-1 were associated with need for admission, and that scores assessed as needing a high intensity bed were significantly higher than those assessed as needing a general bed. There was a linear relationship between the total score and need for admission. The most similar studies to our own for the purpose of comparison are those carried out by Flynn et al. [13], and Freestone et al. [24] in which they analysed the DUNDRUM-1 scores of mainly male patients accepted onto the waiting list for admission to a forensic hospital. The receiver operating characteristic we found in our study for the DUNDRUM-1 of AUC 0.75 (0.63–0.88) was very similar to the AUC of 0.79 [0.67–0.90] and 0.79 [0.72–0.85] found in the studies by Flynn and Freestone respectively. This is consistent with the purpose of the tool, and our finding provides support for the use of this tool in a female population. We also found that higher scores on DUNDRUM-2 were associated with need for admission, and that our AUC score was similar and slightly higher than that reported by Flynn (0.84, 95% CI 0.74–0.94 in our study compared with 0.76, 95% CI 0.63–0.88 in Flynn’s study). The AUC for those actually admitted to hospital was higher in this study, as it was in another study by Flynn et al. [12], indicating the likely prioritisation of those with the greatest need. Although DUNDRUM-2 did not distinguish between need for a general hospital bed versus a high intensity bed, this was not unexpected as DUNDRUM-2 assesses urgency of admission, not security level needed. It was beyond the scope of our study to identify and investigate the scores of those who were actually admitted to hospital, but we would expect that in services where demand exceeds service capacity, where there is need for prioritisation of admission based on severity and acuity of need, that AUC scores of those admitted compared to those assessed as needing admission but not admitted would be higher than those found in our study. We found that neither seriousness of self-harm, nor immediacy of risk of suicide were associated with assessed need for hospital admission. Although the DUNDRUM authors had previously reported this observation [13] it was perhaps surprising that it was also the case in a female population in which self-harm is more common. It may be that the prevalence of self-harm amongst incarcerated women is high for many reasons, so it does not distinguish well between those requiring assistance in adjusting to custody from those with acute mental illness needing hospitalisation. Neither the decision to admit, nor urgency of admission was associated with self-harm among the female population. The main drivers, as in males, were immediacy and severity of violence, specialist forensic need, risk of absconding, complex needs regarding management of violence and institutional behaviour.

### Limitations

There are a number of limitations in our study. First, inmates who were not identified as having mental health needs by healthcare managers would not have been identified during this study, and identification of level of need on the 5-point scale used in this study had not previously been validated. There is therefore a possibility that our study has underestimated the true number that required admission. Another limitation of this study is that only a sample of those categorised as “Moderate” was examined. We anticipated that only cases categorised as “Serious” or “Very Serious” would contain cases that required admission, and that no, or very few cases categorised by the host institutions as “Moderate” would require admission. Contrary to this expectation, 4 out of 16 “Moderate” cases were assessed to require admission. We have extrapolated this proportion across all of those categorised as “Moderate”, however the true number requiring admission in this category may be different. Further, it is possible that some categorised as “Mild” or “No Mental Disorder” were misclassified, such that some of those would also require admission. Overall, it is likely that if there is any error, it would most likely be in the direction of underestimation rather than overestimation of need. Another limitation of this study is that the assessment of whether hospital admission was required was made by the psychiatrists, and were not independent of the rating of DUNDRUM. DUNDRUM ratings made by raters independent to the decision of whether to admit would have provided less chance of bias, however the process in our study was carried out in a clinically realistic process in which the same clinician rated the DUNDRUM and provided an opinion as to whether the inmate needed admission.

## Conclusions

This is the first study to determine level of need for prison to hospital transfers in Canada and can be used to inform service capacity planning. We found that the DUNDRUM toolkit is useful in determining the threshold and priorities for hospital transfer of female prisoners. The strengths of this study are that we have surveyed a complete provincial female prison population in the most populous province in Canada to estimate need for hospital admission. As far as we are aware, such a study has not been done for a female population outside the UK before. We have also provided validation, for the first time, of DUNDRUM in an exclusively female prison sample. As services are scarce and finite, tools and processes that accurately identify those in need are important. The DUNDRUM 1 and 2 tools appear useful triage tools to assist in this assessment and prioritisation of need. Further work is required to validate these findings in other populations and to study outcomes for inmates found to have such high level of mental health need.

## Abbreviations

AUC: Area under the curve
BJMHS: Brief Jail Mental Health Screen
MCSCS: Ministry of Community Safety and Correctional Services
OR: Odds ratio
